# A real-world based study for immunogenicity and safety for three immunization schedules of polio vaccine

**DOI:** 10.1038/s41598-025-89852-x

**Published:** 2025-02-20

**Authors:** Li Sun, Shi-fan Wang, Yi-qing Zhu, Ya-fei Wang, Jun-mian Zhang, Jing-hui Wang, Yan-li Cong, Jing Li, Xiao-qin Liu, Sha-sha Han, Yu Guo, Qi Li

**Affiliations:** 1 Hebei Provincial Center for Disease Control and Prevention, shijiazhuang, 050021 Hebei China; 2 North China University of Science and Technology, Tangshan, 063210 Hebei China; 3 China National Biotec Group Company Limited, Beijing, China

**Keywords:** Polio vaccine, Immunogenicity, Safety, Active monitoring, Passive monitoring, Vaccines, Epidemiology

## Abstract

To evaluate the immunogencity and safety for three immunization schedules of inactivated poliovirus vaccine (IPV) and bivalent oral poliovirus vaccine (bOPV) for providing a basis for further optimization of the polio sequential immunization schedule. To obtain immunogenicity data and to active surveillance the occurrence of adverse events following immunization (AEFI), healthy infants ≥ 2 months of age were randomly chosen in Hebei Province, and were divided into three groups to be vaccinated with IPV-bOPV-bOPV(Group a), IPV-IPV-bOPV(Group b) and IPV-IPV-IPV(Group c) at 2, 3 and 4 months of age respectively. AEFI cases related to poliomyelitis vaccines in Hebei province by passive surveillance from January 1, 2018 to December 31, 2022 were obtained from national adverse event following immunization surveillance system (NAEFISS). After basic immunization with polio vaccine, the positive conversion rate of neutralizing antibodies of types I, II and III were all > 97.00% and the positive rates were all > 98.00%, the geometric mean titer (GMT) was significantly higher than that before basic immunization, the GMT level of neutralizing poliovirus antibody after basic immunization was the highest in type I, followed by type III, and the lowest in type II. A total of 16 AEFI cases (2.52%) were reported by active surveillance, and 2903 AEFI cases (1.40%) were reported by passive surveillance. AEFI reported by both monitoring modalities were dominated by fever of common vaccine reactions. No rare serious adverse reactions like VAPP etc. were monitored and the overall regression was positive. All three immunization schedules for polio vaccine have demonstrated good immunogenicity and safety when administered to healthy populations.

## Introduction

In September 2015, the Global Polio Eradication Certification Committee announced the eradication of wild poliovirus type 2 (WPV2), prompting the World Health Organization (WHO) Strategic Advisory Committee to recommend a global discontinuation of using the type 2 polio vaccine component of oral poliovirus vaccine (OPV). In May 2016, 155 countries around the world, including China, simultaneously implemented a change in polio immunization strategies. At the same time, in response to the call from the World Health Organization (WHO) and the China government, Hebei Province also adjusted the vaccination strategy for polio vaccine. They ceased the use of trivalent OPV (tOPV) in their sequential immunization schedule and began using only bivalent OPV (bOPV) against poliovirus types 1 and 3 (PV1 and PV3) to prevent and control polio. Importantly, they also prioritized including at least one dose of inactivated polio vaccine (IPV) in their sequential immunization schedule to, as much as possible, avoid vaccine-associated paralytic poliomyelitis and circulating vaccine-derived poliovirus (cVDPV) caused by poliovirus types 2 (PV2) in the live polio vaccine while maintaining immunity to PV2, thus minimizing the potential risk of PV2 outbreaks^1–5^. Since January 2020, the routine polio vaccination program in Hebei Province has been adjusted to two doses of IPV followed by two doses of bOPV, that is, infants receive 1 dose of IPV at 2 months of age and 3 months of age respectively, and 1 dose of bOPV at 4 months of age and 4 years old respectively. The bOPV and IPV used in Hebei Province are both included in the Expanded Program on Immunization (EPI) schedule.

Several studies have shown that the revised sequential polio immunization program combines the advantages of bOPV and IPV to reduce the incidence of vaccine associated paralytic poliomyelitis (VAPP) and to produce higher immunity against poliovirus^6–9^.The analysis conducted in this paper aimed to evaluate the immunogenicity and safety for three immunization schedules of polio vaccine, and to provide a basis for continuous improvement of the polio sequential immunization program.

## Methods

### Sample size

Based on the Phase III clinical trial data for IPV from Beijing Institute of Biological Products Co., Ltd., positive conversion rate for Type I, Type II, and Type III antibodies were 96.2%, 93.8%, and 97.6%, respectively. The lowest positive conversion rate of 93.8% was selected as an indicator. Employing the formula $N = \left( {\frac{{Z_{{\left( {1 - \alpha } \right)}} + Z_{{\left( {1 - \beta } \right)}} }}{\delta }} \right)^{2} \cdot \pi _{0} \cdot \left( {1 - \pi _{0} } \right)$ (α = 0.05, β = 0.01) to calculate, the calculated sample size was determined to be 50. Accounting for a dropout rate of 20%, the sample size was rounded up to 63, ensuring that the sample size per group is no less than 63 individuals.

### Research program

**Fig. 1:**
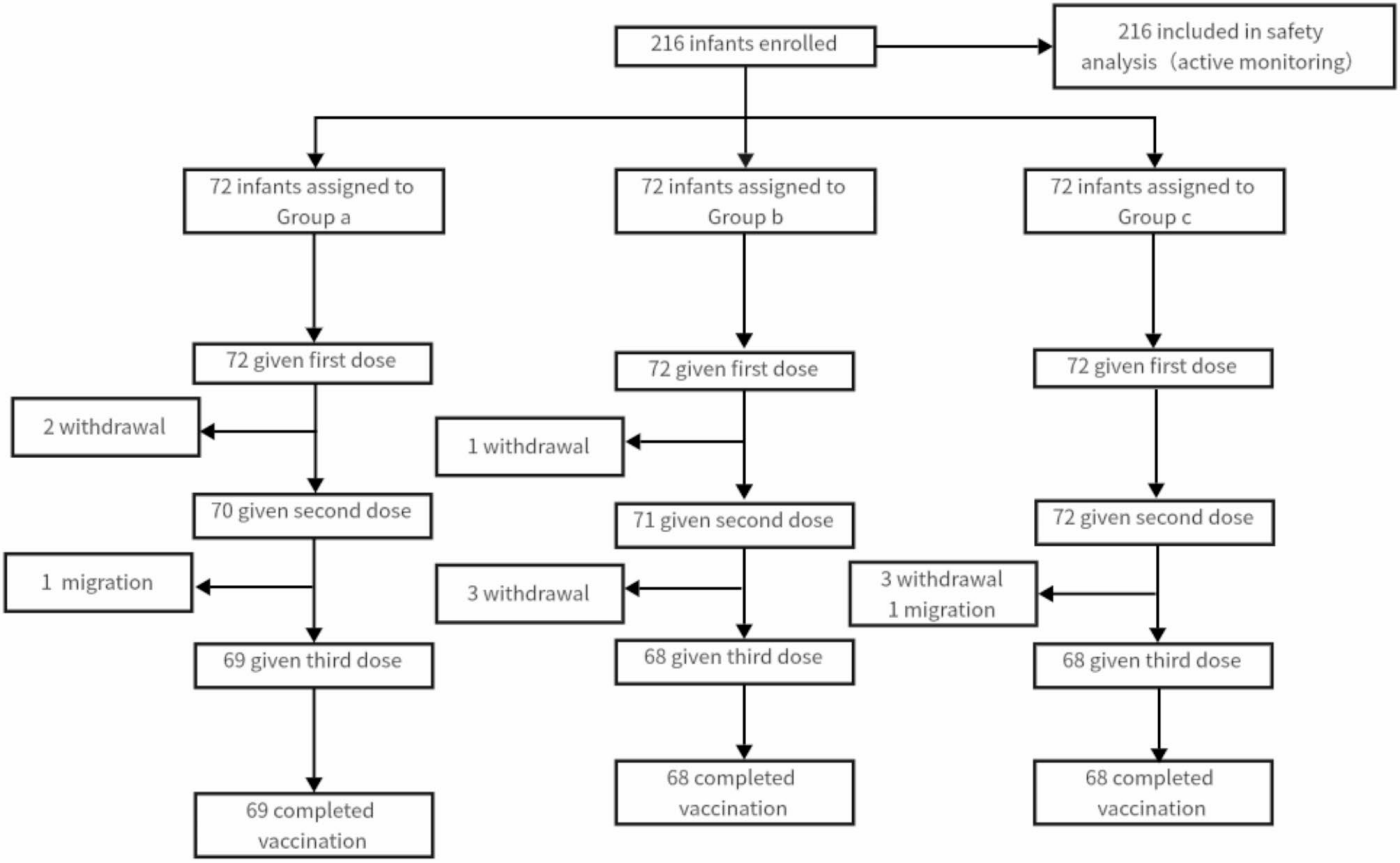
Programme profile.

The study was conducted a vaccination clinic in Hebei Province from June 1st to December 30th in 2018. Infants were included based on the following criteria: ① Age ≥ 2 months on the day of enrollment; ② The guardians of infants signed an informed consent form; ③ The guardians were able to participate in all follow-up plans and adhere to all research procedures; ④ The interval of the most recent vaccination was ≥ 14 days; ⑤ Healthy infants with a body temperature ≤ 37 °C, who had been physically examined and had their health status inquired by a clinical physician before entering the project. There were 216 individuals have provided their consent to participate in the experiment by signing the informed consent documents, and all of them have been enrolled in the study. Infants meeting the inclusion criteria were randomly divided into three immunization program groups: IPV-bOPV-bOPV (Group a), IPV-IPV-bOPV (Group b), and IPV-IPV-IPV (Group c), with 72 in each of groups a, b, and c. Eventually, due to the occurrence of withdrawl and migration, the number of infants who ultimately persevered through the study in the 3 groups was 69, 68, and 68, respectively. Active safety monitoring following polio vaccine administration was conducted on all 216 infants (Fig. 1).The IPV and bOPV used in this research were both manufactured by Beijing Institute of Biological Products Co., Ltd., and were within their respective expiration dates. IPV: Liquid, each vial contains 0.5 mL; bOPV: Liquid, each vial contains 1.0 mL (for 10 doses).

### Immunogenicity study

1. Vaccination procedure
  The three groups of infants received their first dose of the vaccine at 2 months of age (60–89 days post-birth), the second dose at 3 months of age (90–119 days post-birth), and the third dose at 4 months of age (120–149 days post-birth), with a 30-day interval between each vaccination.
2. Serum sample collection
  Serum samples were collected at two time points: prior to the administration of the first dose of the vaccine at 2 months of age of infants, and 30 days after the administration of the third dose of the vaccine.
3. Study endpoint
  This study is a non-inferiority study to evaluate the immunogenicity of different immunization schedules for polio vaccine, with the primary endpoint of positive conversion rate and antibody levels 30 days after the completion of vaccination procedure.
4. Testing institutions and methods
  The detection of Type I and Type III poliovirus neutralizing antibodies was completed by the Polio Network Laboratory of the Beijing Center for Disease Prevention and Control. The detection of Type II poliovirus neutralizing antibodies was completed by Chinese Center for Disease Control and Prevention (CDC) using the micro-neutralization test to determine the poliovirus neutralizing antibodies. The tested serum was diluted fourfold with a nutrient solution prepared from cell culture medium (Minimum Eagle’s Medium, MEM) and inactivated at 56 °C in a water bath for 30 min, followed by further serum dilution. In a 96-well microtiter plate, 50 µl of MEM nutrient solution was added to each well, and inactivated serum samples (fourfold) were added to wells A1 to H1 (50 µl per well), with each serum sample added to two wells. Four samples of serum could be added per plate. Following this method, three plates were prepared, labeled for Type I, Type II, and Type III, respectively. A 12-channel pipette was used to mix from well A1 to H1, then 50 µl was drawn and serially diluted to wells A12 to H12, discarding 50 µl. Each batch of experiments included a virus control, a standard serum control, and a cell blank control, ensuring that the titer of the challenge virus was 100 CCID 50/0.05 ml and the cell suspension concentration was between 1.0 × 10^5^ and 2.5 × 10^5^ cells/ml.
5. Immunogenicity indicators
  A neutralizing antibody titer of ≥ 1:8 is considered positive, indicating a protective effect against the poliovirus; a titer of < 1:8 before basic immunization and ≥ 1:8 after is considered a seroconversion, or an increase of 4-fold or more after basic immunization compared to before is also considered a seroconversion; poliovirus neutralizing antibody titers of < 8 are calculated as 4, and titers > 16,384 are calculated as 32,768 to determine the Geometric Mean Titer (GMT).

### Safety Surveillance

1. Active monitoring
  All infants were observed on-site for 30 min after vaccination to collect and record any AEFI.
  Daily telephone follow-ups were conducted for seven consecutive days after each vaccine dose. Subsequently, a telephone follow-up was performed once a week until the next vaccination dose was administered. Referring to the literature^10^, fever was categorized as follows: (1) Low-grade fever (≤ 38 °C); (2) Moderate fever (38.1–39 °C); (3) High fever (39.1–41 °C); (4) Extremely high fever (≥ 41 °C).
2. Passive monitoring
  Data for AEFI reported in Hebei Province during the 3-year period, from January 2018 to December 2022, was collected through the NAEFISS. The yearly number of distributed doses of various vaccines in Hebei province during the study period was obtained from China Immunization Programming Information Management System and Vaccination Data Sheet.
  AEFI are classified into vaccine product-related reaction (includes common vaccine reactions and rare vaccine reactions), vaccine quality incident, program error, coincidental event and psychogenic reaction. According to severity, AEFI needs to be categorized into severe AEFI and non-severe AEFI. Each AEFI record needs to be followed up by the county-level CDC staff and mark the outcome of patients as cure, improved, required treatment, condition deteriorates, sequelae or death.

### Statistics analysis

A database was organized as an Excel file (Microsoft Office Excel 2010), statistical analysis were performed by Stata 14.0 and SPSS 21.0. Quantitative data that were normally distributed are presented as mean ± standard deviation (‾x ± s), and group comparisons were made using analysis of variance (ANOVA). Quantitative data not normally distributed are represented by median and interquartile range [M(Q1, Q3)], with pre- and post-immunization GMT comparisons made using the Mann-Whitney U test. Group comparisons were conducted using the Kruskal-Wallis H test, and pairwise group comparisons were performed using the Nemenyi method. Categorical data were analyzed using the chi-square test and Fisher’s exact probability method, with a significance level of α = 0.05. Descriptive analysis was conducted for AEFI indicators, with the reporting rate of an AEFI for a vaccine (100 doses) = the number of AEFI reports / the number of vaccinations×100 doses.

### Statement

All methods were performed in accordance with the relevant guidelines and regulations.

## Results

### Immunogenicity study

#### Positive rate for neutralizing antibody and positive conversion rate

Before immunization, there was no statistically significant difference in the positive rates for neutralizing antibodies among all types in groups a, b, and c (*P* > 0.05), indicating that the levels of neutralizing antibodies before immunization were comparable across groups. After immunization, the positive rates for neutralizing antibodies for each type in all groups ranged from 98.53 to 100.00%, and the positive conversion rates ranged from 97.06 to 100.00%. There were no statistically significant differences in positive conversion rates among the groups for each type of neutralizing antibody (*P* > 0.05) (Table 1).

**Table 1:** Comparison of positive rate for neutralizing antibody and positive conversion rate before and after immunization.

Serotype	Parameter	Group a (*N* = 69)		Group b (*N* = 68)		Group c (*N* = 68)		*χ* ^2^	*P*
		*N*	%	*N*	%	*N*	%		
Type I									
Before	Positive rate	26	37.68	35	51.47	25	37.76	3.798	0.150
After	Positive rate	69	100.00	68	100.00	68	100.00	-	-
	Positive conversion rate	43	100.00	33	100.00	43	100.00	3.793	0.150
Type II									
Before	Positive rate	35	50.72	35	51.47	25	37.76	3.761	0.153
After	Positive rate	68	98.55	68	100.00	67	98.53	-	-
	Positive conversion rate	33	97.06	33	100.00	42	97.67	3.373	0.185
Type III									
Before	Positive rate	7	10.14	11	16.18	11	16.18	1.371	0.504
A fter	Positive rate	69	100.00	68	100.00	68	100.00	-	-
	Positive conversion rate	62	100.00	57	100.00	57	100.00	1.438	0.487

#### The GMT levels of neutralizing antibodies

Before and after immunization for groups a, b, and c, and for types I to III, all showed statistically significant differences (*P* < 0.05). For type I, the differences between groups a and b, and between b and c were statistically significant. For type II, the differences between groups a and b, and between a and c were statistically significant. For type III, the differences among all groups were statistically significant. After primary immunization, the GMT levels of neutralizing antibodies in all three groups were the highest for type I, followed by type III, and the lowest for type II (Table 2).

**Table 2:** Comparison of the GMT Levels of Neutralizing Antibodies before and after immunization.

Serotype	Parameter	Group a (*N* = 69)		Group b (*N* = 68)		Group c (*N* = 68)		*H*	*P*
		Value	95% CI	Value	95% CI	Value	95% CI		
Type I									
Before	GMT	6.48	5.37−7.82	8.86	6.91−11.35	6.46	5.42−7.69		
After	GMT	4 907.83	3629.47−6 636.45	7175.30	5344.60−9 633.08	4310.17	3481.08−5336.74	16.66	< 0.001
Type II									
Before	GMT	7.92	6.38−9.83	7.84	6.45−9.52	6.87	5.65−8.34		
After	GMT	41.55	30.39−56.82	200.44	148.77−270.06	400.89	296.71−541.64	74.25	< 0.001
Type III									
Before	GMT	4.47	4.11−4.86	4.71	4.27−5.19	4.71	4.24−5.23		
After	GMT	1692.15	1342.06−2133.55	2986.25	2202.06−4049.69	1013.62	752.61−1365.13	32.06	< 0.001

#### Difference in the fold increase of polio neutralizing antibodies

The differences for types II and III between different groups were statistically significant. For type I, there were no statistically significant differences among the three groups; for type II, all three groups showed statistically significant differences, with the GMT fold increase being group c > group b > group a; for type III, there were statistically significant differences between groups a and b, and between group b and c (Table 3).

**Table 3:** Comparison of the fold increase in Polio neutralizing antibodies before and after immunization.

Group	M(Q1, Q3)		
	Type I	Type II	Type III
Group a	1024(256, 2048)	4(2, 16)^#Δ^	512(256, 1024)^#^
Group b	1024(512, 2048)	32(8, 128)^*Δ^	1024(512, 2048)
Group c	512(512, 1024)	64(16, 256)^*#^	256(128, 512)^#^
*H*	4.62	56.53	27.29
*P*	0.099	< 0.001	< 0.001

^*^Compared to group a *P* < 0.05, ^#^Compared to group b *P* < 0.05, ^Δ^Compared to group c *P* < 0.05.

### Safety study by active surveillance

#### The baseline characteristic of AEFI

A total of 16 AEFI cases were reported, with a reporting rate of 2.52%. Group a collected 5 AEFI cases (2.23%), group b collected 2 AEFI cases (0.97%) and group c collected 9 AEFI cases (4.41%). The AEFI reporting rate in group b was lower than that in group c (*χ*^2^ = 4.683, *P* < 0.05).

#### Clinical diagnosis of AEFI

All 16 reported AEFI cases were common vaccine reactions, and presented with fever symptoms. There was 1 case of low-grade fever (0.16%), 11 cases of moderate fever (1.73%), and 4 cases of high fever (0.63%). The AEFI reporting rates for the three types of fever from highest to lowest were as follows: moderate fever, high fever, and low-grade fever (*χ*^2^ = 9.959, *P* < 0.05). No cases of VAPP or VDPV were detected (Table 4).

**Table 4:** Clinical diagnosis of AEFI (n, %).

Group	Low-grade fever		Moderate fever		High fever		Total	
	No. of cases	Rate	No. of cases	Rate	No. of cases	Rate	No. of cases	Rate
Group a	1	0.45	4	1.79	0	0	5	2.23
Group b	0	0	1	0.48	1	0.48	2	0.97
Group c	0	0	6	2.94	3	1.47	9	4.41
Total	1	0.16	11	1.73	4	0.63	16	2.52

#### Time interval from vaccination to onset and outcome distribution

All 16 AEFI cases occurred within 15–30 days post-vaccination, and all have fully recovered.

### Safety study by passive surveillance

#### The baseline characteristic of AEFI cases

From 2018 to 2022, a total of 2,903 AEFI cases were reported and 2,074,772 doses of vaccine were administered (group a: 1,108,960 doses, group b: 864,935 doses, group c: 100,877 doses), with a reported rate of 0.14%. The AEFI cases reported for groups a, b, and c were 1,489 cases (0.13%), 1,275 cases (0.15%), and 139 cases (0.14%), with the rate of AEFI in group b being higher than that in group a (*χ*^2^ = 6.000, *P* < 0.05).

There were 2,837 cases of common vaccine reactions (0.14%), 52 cases of rare vaccine reactions (0.003%), and 14 cases of coincidental events (0.001%); 2,888 (0.14%) non-severe AEFI cases and 15 (0.001%) sever AEFI cases reported after vaccination (Table 5).

**Table 5:** Classification of AEFI (n, %).

	Group a		Group b		Group c		Total	
	No. of cases	Rate	No. of cases	Rate	No. of cases	Rate	No. of cases	Rate
Classification								
Common vaccine reaction	1446	0.130	1255	0.142	136	0.135	2837	0.137
Rare vaccine reaction	34	0.003	16	0.002	2	0.002	52	0.003
Coincidental event	9	0.001	4	0.001	1	0.001	14	0.001
Severity								
Non-severe	1480	0.130	1270	0.147	138	0.137	2888	0.139
Sever	9	0.001	5	0.001	1	0.001	15	0.001
Total	1489	0.134	1275	0.147	139	0.138	2903	0.140

#### Clinical diagnosis of common and rare vaccine reactions

Among the 2,837 cases of common vaccine reactions, fever were predominant, accounting for 2,261 cases (0.11%), followed by local redness and induration with 598 cases (0.03%) and 250 cases (0.01%). The reported rate of fever in Group b was higher than that in Group a (*χ*^2^ = 9.361, *P* < 0.05).

Among the 52 cases of rare vaccine reactions, allergic reactions were the main type with 34 cases (0.002%), primarily consisting of allergic rashes with 21 cases (0.001%), followed by urticaria with 8 cases (0.001%). The reported rates of allergic reactions and other clinical diagnoses in the three groups showed no statistical significance (*P* > 0.05). No cases of vaccine-derived poliovirus (VDPV) or vaccine-associated paralytic poliomyelitis (VAPP) were detected. (Table 6).

**Table 6:** Clinical diagnosis of common and rare vaccine reactions (n, %).

Clinical diagnosis	Group a		Group b		Group c		Total	
	No. of cases	Rate	No. of cases	Rate	No. of cases	Rate	No. of cases	Rate
Common vaccine reaction								
Fever (°C)	1137	0.103	1012	0.117	112	0.111	2261	0.109
Redness (cm)	287	0.026	290	0.034	21	0.021	598	0.029
Induration (cm)	108	0.010	129	0.015	13	0.013	250	0.012
Rare vaccine reaction								
Allergic reaction								
Allergic skin rash	12	0.001	8	0.001	1	0.001	21	0.001
Nettle rash	6	0.001	2	0.001	-	-	8	0.001
Maculopapule	2	0.001	-	-	-	-	2	0.001
Angioedema	2	0.001	-	-	-	-	2	0.001
Measles scarlet fever-like rash	1	0.001	-	-	-	-	1	0.001
Total	23	0.002	10	0.001	1	0.001	34	0.002
Epilepsy	1	0.001	-	-	1	0.001	2	0.001
Encephalopathy	1	0.001	1	0.001	-	-	2	0.001
Sterile abscess	1	0.001	1	0.001	-	-	2	0.001
Thrombocytopenic purpura	3	0.001	2	0.001	-	-	5	0.001
Febrile convulsion	1	0.001	1	0.001	-	-	2	0.001
Others	4	0.001	1	0.001	-	-	5	0.001

#### Interval between vaccination and onset

There were 2,122 AEFI cases (73.10%) occurred < 1 d after vaccination and 781 AEFI cases (26.90%) occurred ≥ 1 d. Among 2,837 cases of common vaccine reactions, the number of cases occurring < 1 d, 1−3 d, 4−14 d, ≥ 15 d after vaccination were 2,079 (73.28%), 719 (25.34%), 37 (1.30%), and 2 (0.08%). Among 52 cases of rare vaccine reactions, the number of cases occurring < 1 d, 1−3 d, 4−14 d, ≥ 15 d after vaccination were 33 (63.46%), 14 (26.92%), 3 (5.77%) and 2 (3.85%).

### Outcome distribution

2,790 cases (96.11%) resulted in cured and improved, 111 cases (3.82%) of requiring treatment, and 2 cases (0.07%) of death (both were coincidental events).

## Comparative analysis of active and passive surveillance

The reporting rates of AEFI and common vaccine reaction in active surveillance were higher than those in passive surveillance (*χ*^2^ = 239.307, 314.988, *P* < 0.05), while there was no statistically significant difference in the reporting rates of rare vaccine reactions between the two surveillance methods.

## Discussion

Since the goal of eradicating polio was set in 1988 and the Global Polio Eradication Initiative was launched worldwide, there has been a significant decline in polio cases globally, making polio the second infectious disease after smallpox that humanity hopes to eradicate^10^. As of 2018, out of 194 countries/regions worldwide, 69 have included IPV in their national immunization programs, with 50 countries/regions adopting a full course of IPV, and 19 countries/regions using a strategy of administering IPV for the first 1–2 doses, followed by bOPV^11^. This exploratory study could provide scientific data for future policy changes in China.

In this study, the GMT of all types and all groups significantly increased compared to before the basic immunization, with the highest GMT levels for Type I, followed by Type III and the lowest for Type II. The reason might be that the first dose of all three vaccination schedules was IPV, which eliminated the interference and impact of Type II polio vaccine virus on Types I and III^12^. The positive rate for neutralizing antibody and positive conversion rate for Types I, II, and III in all three groups were above 97.06%. However, the GMT and positive conversion rate for Type II antibodies were highest in Group c, followed by Group b and Group a, consistent with the findings of Hui Ye et al.^13^ and Qian Li et al.^14^. The positive rate for neutralizing antibody for Types I and III in Groups a and Group b both reached 100.00%, in line with the results of Gao S et al.^15^. The three immunization schedules were effective after the basic immunization, providing good immune protection against all three polio types, consistent with the results of related studies^16,17^.

Active surveillance data indicates that the reporting rates of AEFI for the three immunization schedules are higher than those reported in China (0.03–0.1%)^18^, Hebei (0.01–0.06%)^19^, and Jilin Province (0.04–0.17%)^20^. This could be attributed to the higher sensitivity of active surveillance, which allows for the timely acquisition of detailed information on AEFI occurrences. All AEFI cases were common vaccine reactions with moderate fever being the main type. All AEFI cases occurred within 15–30 days after vaccination, which differs from the finding reported in Jiangxi Province (mainly occurred within 2days after vaccination)^21^. The specific reasons for this discrepancy warrant further investigation. No rare serious AEFI were detected during study period, and all cases have been cured.

Passive surveillance data indicates that the overall reporting rate of AEFI is significantly higher than the findings reported in Guangxi Province (0.003%)^22^ and Shaanxi Province (0.01%)^23^. This may be attributed to the incident of failure in the evaluation of the potency of acellular pertussis combined vaccine and the irregular production of rabies vaccines for human happened in 2018 which have likely heightened public awareness and concern about vaccine safety and AEFI, leading to a stronger willingness to report adverse events after vaccination. The common vaccine reactions are primarily fever and localized reactions at the injection site which are mild. Rare vaccine reactions are predominantly allergic reactions, possibly caused by the use of excipients or adjuvants, including magnesium chloride, during the production of bOPV. These reactions are mostly concentrated within one day after vaccination, with a generally positive outcome. This also suggests that caregivers should carefully inquire about the child’s allergy history and other relevant information from the guardian before vaccination. Additionally, no cases of VAPP or VDVP have been detected.

Passive surveillance reports a significantly lower rate of AEFI and common vaccine reactions compared to active surveillance. On one hand, this may be attributed to the fact that vaccinees may neglect minor reactions and choose not to report them, thus making these cases undetectable by passive surveillance. On the other hand, current passive surveillance primarily relies on voluntary reporting by the parents and then reports are filled out and submitted by personnel from healthcare facility, CDC at any administrative levels, and vaccination units. Consequently, the reporting rate is influenced by multiple factors. Given various limitations, passive surveillance remains the primary method for monitoring the safety of post-marketing vaccines.

The limitation of this study may include the following aspects. Firstly, the period of active surveillance is quite short, but we do our best to ensure that all researchers have standardized training and are able to guarantee the accuracy of the data. Secondly, for passive reporting system, number of mild AEFI may be easily neglected by healthcare workers and/or vaccinees to report resulting in underestimation of AEFI incidence rate.

## Conclusion

All three immunization schedules for polio vaccine have demonstrated good immunogenicity and safety when administered to healthy populations. In particular, immunogenicity for Type II antibody was better when administering 3 doses of IPV than 2IPV + 1bOPV and 1IPV + 2bOPV program. Referring to the WHO’s recommendation, Hebei Province can gradually transition to a full IPV vaccination strategy considering the national policy and actual occurrence of poliomyelitis in neighboring countries.

## Data Availability

The data that support the findings of this study are available from the corresponding author (Yu Guo) upon reasonable request.
